# Modifications of EEG power spectra in mesial temporal lobe during *n*-back tasks of increasing difficulty. A sLORETA study

**DOI:** 10.3389/fnhum.2013.00109

**Published:** 2013-04-02

**Authors:** Claudio Imperatori, Benedetto Farina, Riccardo Brunetti, Valentina Gnoni, Elisa Testani, Maria I. Quintiliani, Claudia Del Gatto, Allegra Indraccolo, Anna Contardi, Anna M. Speranza, Giacomo Della Marca

**Affiliations:** ^1^Department of Human Science, European University of RomeRome, Italy; ^2^Department of Neurosciences, Catholic UniversityRome, Italy; ^3^Department of Dynamic and Clinical Psychology, Sapienza UniversityRome, Italy

**Keywords:** EEG, LORETA, mesial temporal lobe, *n*-back, working memory

## Abstract

The *n*-back task is widely used to investigate the neural basis of Working Memory (WM) processes. The principal aim of this study was to explore and compare the EEG power spectra during two *n*-back tests with different levels of difficulty (1-back vs. 3-back). Fourteen healthy subjects were enrolled (seven men and seven women, mean age 31.21 ± 7.05 years, range: 23–48). EEG was recorded while performing the *N-back* test, by means of 19 surface electrodes referred to joint mastoids. EEG analysis were conducted by means of the standardized Low Resolution brain Electric Tomography (sLORETA) software. The statistical comparison between EEG power spectra in the two conditions was performed using paired t-statistics on the coherence values after Fisher's z transformation available in the LORETA program package. The frequency bands considered were: delta (0.5–4 Hz); theta (4.5–7.5 Hz); alpha (8–12.5 Hz); beta (13–30 Hz); gamma (30.5–100 Hz). Significant changes occurred in the delta band: in the 3-back condition an increased delta power was localized in a brain region corresponding to the Brodmann Area (BA) 28 in the left posterior entorhinal cortex (*T* = 3.112; *p* < 0.05) and in the BA 35 in the left perirhinal cortex in the parahippocampal gyrus (*T* = 2.876; *p* < 0.05). No significant differences were observed in the right hemisphere and in the alpha, theta, beta, and gamma frequency bands. Our results indicate that the most prominent modification induced by the increased complexity of the task occur in the mesial left temporal lobe structures.

## Introduction

Over the last 30 years the concept of working memory (WM) has been investigated extensively, from cognitive psychology to neuroscience (Baddeley, 2010, 2011). WM can't be defined unequivocally, because it is presently described as emerging from the interplay of multiple cognitive functions (i.e., attentional and mnemonic functions) with different neural bases (Bledowski et al., 2010). Several studies have investigated the neurological basis of WM (Owen et al., 2005; Muller and Knight, 2006; Schlosser et al., 2006; Linden, 2007) confirming the multiple nature of WM and the involvement of different areas such as ventral and dorsal lateral prefrontal cortex, parietal and temporal lobes. On the basis of neuroimaging and functional studies, a multicomponent WM model has been proposed (Baddeley, 2000). According to this model, a “phonological loop” and a “visuo-spatial sketchpad” constitute a slave systems that can independently process different types of information. This information coming from different sources is integrated by a unitary control mechanism, which is named “the central executive.” Both are linked to long-term memory via an episodic buffer. A revision of the literature about lesion studies (Muller and Knight, 2006) suggests a functional dichotomy of the visuo-spatial sketchpad: ventral occipito-temporal area is involved in the object recognition while the dorsal connection between occipital and parietal cortices is crucial for spatial relation. Furthermore, lesion studies (Muller and Knight, 2006) indicate that the phonological loop can be divided in a phonological short-term store and an articulatory subvocal rehearsal system, respectively, localized in inferior parietal cortex and in the brain areas necessary for speech production (i.e., Broca's area, the supplementary motor association area and possibly the cerebellum). The anatomical base of central executive is more uncertain. A link between this system and the prefrontal area is widely documented (D'Esposito et al., 1995; Salmon et al., 1996; Collette et al., 1999). Muller e Knight (Muller and Knight, 2006) have proposed that the central executive is distributed along ventral and dorsal lateral prefrontal cortices. Nevertheless, it has been argued (Andres, 2003) that a more complex and dynamic view of its neural substrate is needed; for example, the central executive system does not include exclusively the frontal cortex, but it involves different brain areas, such as the parietal areas (Garavan et al., 2000) and the anterior cingulated cortex (D'Esposito et al., 1995).

In the last decades the *n*-back task has been widely used to investigate the neural basis of WM processes (Braver et al., 1997; McEvoy et al., 1998; Jansma et al., 2000; Kane and Engle, 2002; Ragland et al., 2002; Conway et al., 2005; Ravizza et al., 2005). In this task, subjects identify over consecutive trials whether the current stimulus (generally letters or numbers, but also emotional stimuli) matches a stimulus presented *n* trials previously (Chatham et al., 2011), usually 1, 2, or 3; the load factor *n* can be adjusted to make the task more or less difficult with consequential changes in brain activity (Braver et al., 1997; Druzgal and D'Esposito, 2001; Honey et al., 2002; Pesonen et al., 2007).

Owen and coworkers (2005) have conducted a detailed meta-analysis of the neuroimaging *n*-back studies. Authors reported that six cortical regions were defined as consistently activated: essentially, the prefrontal and premotor cortices, the cingulated cortex, the medial posterior parietal cortex, and the cerebellum. Other studies (Gevins et al., 1997; Gevins and Smith, 2000; Deiber et al., 2007; Lei and Roetting, 2011; Palomaki et al., 2012) have evaluated the memory load manipulations in *n*-back task by means of scalp EEG recordings and have reported that modification linked to the complexity of the WM task occurred prevalently in the alpha and theta frequency bands especially in frontal and parietal lobes. In particular, increased task difficulty (which means increased memory load) was associated with increased theta band power in the frontal midline electrodes which means increased frontal midline theta activity (Gevins et al., 1997). This finding was confirmed in other studies (Gevins and Smith, 2000; Lei and Roetting, 2011). Frontal midline theta (Onton et al., 2005), like hippocampal theta, plays a role in processing of memory and emotion, and it is correlated with WM and or sustained attention (Mitchell et al., 2008). Together with modifications in theta frequency, most Authors observed modification in the alpha power and alpha frequency. In the study by Gevins et al. (1997), increased task difficulty was associated with reduction of the slow frequency alpha rhythm (8.5–10 Hz) in the parieto-occipital midline areas; whereas the fast-frequency alpha band (10–13.5 Hz) showed a task-dependent behavior: it was less reduced in the verbal than in the spatial task (Gevins et al., 1997). Conversely, other Authors reported a reduction of alpha band power (Lei and Roetting, 2011). It has been hypothesized that the amplitude of posterior alpha-band oscillations during short-term memory retention reflects a mechanism that protects fragile short-term memory retention activations from interference by gating bottom-up sensory inputs (Payne and Kounios, 2009). However, in these research not all frequency bands were considered: in particular, the delta EEG frequency band (>4 Hz) was not analyzed in all the studies previously reported.

The principal aim of this study was to explore and compare the EEG power spectra for all frequency bands, including delta (0.5–4 Hz) and gamma (30.5–100 Hz), during two *n*-back tests with different levels of difficulty: 1-back vs. 3-back.

In order to detect modification of EEG frequencies, and their topographic distribution, we used the standardized Low Resolution brain Electric Tomography (sLORETA) software, a validated method for localizing the electric activity in the brain based on multichannel surface EEG recordings (Pascual-Marqui et al., 1994).

## Materials and methods

### Subjects

Fourteen healthy subjects were enrolled in the study (seven men and seven women, mean age 31.21 ± 7.05 years, range: 23–48). All subjects were right-handed, and they were asked to retain from any central nervous system active drugs in the 2 weeks before the study. The local ethical committee approved the study and participants gave their written consent to participate.

### N-back task

The *n*-back test was performed according to a modified version of the standardized methodology (Braver et al., 1997). The experimental procedure was a visual sequential letter memory task with two different memory load (1-back vs. 3-back). Visual stimuli consisted in random sequences of letters, presented black on white background. *n*-back task was displayed on a computer screen (screen 36.4 × 24.9; resolution 1440 × 900). The size of the letters was 4.5 × 4.5 (visual angle: 5.38). Participants were at 50 cm (visual angle: 51) from the screen. The computer was running a custom-made patch in the run time version of Max 6.1 (Cycling'74). Subjects were instructed to press the space bar to indicate the target stimulus; no response was requested for non-target stimuli. The target in the 1-back condition consisted in a letter identical to the immediately preceding one; while the target stimulus in the 3-back condition consisted in a letter identical to the letter presented three trials back. 1- and 3-back conditions consisted in 90 trials each. Trials in the *n*-back consist of one letter each in a continuous flows of displayed letters. The presentation of each letter lasted 500 ms. The interval between the visual letters presented was 1500 ms in both the 1-back and the 3-back conditions tested. In this period participants saw a fixation point. In the 1-back condition, the participants were asked to press the spacebar when the current letter matched that one presented before (2 s); in the 3-back condition, the participant was asked to press the spacebar when the current letter matched the letters presented three letters previously (6 s). The more difficult 3-back session was performed 15 min after the 1-back.

Before 1-back and 3-back task there was a brief training session. The participants were instructed to respond as fast and as accurately as they can to each stimulus by pressing one button for targets and no response for non-targets. We categorized four different kind of possible answers: Hit, Miss, False Alarm (FA), and Correct Rejection (CR). Also reaction time (RT) was calculated for each response.

### EEG recordings

EEG was recorded by means of a Micormed SystemPlus digital EEGraph. EEG recordings were performed continuously during the administration of *n*-back. Recordings were performed in the EEG lab, subjects were sitting in a comfortable armchair, in front of a PC monitor, on which the *n*-back task was performed. EEG montage included 19 standard scalp leads positioned according to the 10–20 system (recording sites: Fp1, Fp2, F7, F3, Fz, F4, F8, T3, C3, Cz, C4, T4, T5, P3, Pz, P4, T6, O1, O2), EOG and EKG. The reference electrodes were placed on the linked mastoids. Impedances were kept below 5 KΩ before starting the recording and checked again at the end. In particular, impedances of the mastoids reference electrodes were checked to be identical. Sampling frequency was 256 Hz; A/D conversion was made at 16 bit; pre-amplifiers amplitude range was ±3200 mV and low-frequency pre-filters were set at 0.15 Hz. In the off-line analysis, artifact rejection was performed visually. Artifact rejection (eye movements, blinks, muscular activations, or movement artifacts) was performed on the raw EEG trace, by posing a marker at the onset of the artifact signal and a further marker at the end of the artifact. Successively, the artifact segment (that is, the EEG signal interval included between the two markers) was deleted, and this cancellation involved all the EEG traces acquired within that interval. In this way, all the EEG intervals characterized by the presence of artifacts were excluded from the analysis. After artifact rejection, the remaining EEG intervals were exported into American Standard Code for Information Interchange (ASCII) files, and imported into the sLORETA software. At least 120 s (not necessarily consecutive) of EEG recording were analyzed for each condition (1-back and 3-back), in all subjects. The average time analyzed was 157 ± 18 s.

### Frequency analysis

All EEG analysis were performed by means of the sLORETA software (Pascual-Marqui et al., 1994). EEG frequency analysis was performed by means of Fast Fourier Transform algorithm, with a 2 s interval on the EEG signal, in all scalp locations. The following frequency bands were considered: delta (0.5–4 Hz); theta (4.5–7.5 Hz); alpha (8–12.5 Hz); beta (13–30 Hz); gamma (30.5–100 Hz). For Frequency analysis, monopolar EEG traces (each electrode referred to joint mastoids) were used, and non the average reference. Topographic sources of EEG activities were determined by means of the sLORETA software. The sLORETA software computes the current distribution throughout the brain volume. In order to find a solution for the 3-dimensional distribution of the EEG signal, the sLORETA method assumes that neighboring neurons are simultaneously and synchronously activated. This assumption rests on evidence from single cell recordings in the brain that shows strong synchronization of adjacent neurons (Kreiter and Singer, 1992; Murphy et al., 1992). Therefore, the computational task is to select the smoothest of all possible 3-dimensional current distributions, a task that is a common procedure in generalized signal processing (Grave De Peralta-Menendez and Gonzalez-Andino, 1998; Grave De Peralta Menendez et al., 2000). The result is a true 3-dimensional tomography, in which the localization of brain signals is preserved with a low amount of dispersion (Pascual-Marqui et al., 1994). Nevertheless, this assumption constitutes an unavoidable limitation of the methods, since it has been demonstrated that functionally distinct areas can be anatomically close, which is problematic for the assumption of adjacent neuronal sources showing highly correlated activity (Hamalainen, 1995).

In order to further test whether or not the EEG signal was modulated by task difficulty, we also performed a comparison of EEG power spectra recorded at scalp sites. For this comparison, the same power bands used in the sLORETA analysis were considered. Moreover, in order to better evaluate the anatomical localization of EEG modification, scalp electrodes were analyzed in groups, divided in: frontal (Fp1, Fp2, F3, Fz, F4), fronto-parietal (F7, Fz, F8, C3, Cz, C4), parieto-temporal (T3, T4, P3, Pz, P4, T5, T6), and occipital (O1, O2). For those group of electrodes were significant modification were observed, also right vs. left side power spectra were compared in the two conditions (1-back and 3-back).

### Statistical analysis

The scores of each subject in the 1-back and 3-back tests were compared. We analyzed: mean sensitivity measures (A′), mean decision criterion (B″d), and mean RT. Comparison was performed by ANOVA analysis. *n*-back statistics were performed by means of the SYSTAT 12 software version 12.02.00 for windows (copyright SYSTAT® Software Inc. 2007).

The statistical comparison of power spectra was performed by means of the LORETA statistical tool (Pascual-Marqui et al., 1994). The power of each frequency band was performed in the two conditions: 1-back vs. 3-back. Correction of significance for multiple testing was computed for the two comparisons between conditions for each frequency band: for the correction, we applied the non-parametric randomization procedure available in the sLORETA program package (Nichols and Holmes, 2002).

To further test whether or not the EEG signal was modulated by task difficulty we perform frequency power analyses at scalp sites using a non-parametric Mann–Whitney *U*-test (due to the non-normal distribution of the power spectral values, as performed by Shapiro–Wilk test *p* < 0.001). In case of multiples comparison, in order to avoid family-wise type-I errors, a formal Bonferroni correction was applied to each family of comparisons, by dividing the limit of significance by the number of comparisons (for frequency analysis six comparisons were made, in the conditions delta, theta, alpha, beta, gamma; therefore the threshold level for significance was *p* = 0.05/6 = 0.008).

## Results

### N-back behavioral results

All subjects completed the two conditions of the *n*-back task, in a within design. Subjects obtained lower scores in the 3-back in the sensitivity measures (A′) and in a decision criterion (B″d), compared to the 1-back condition (see Table 1). The difference between the 1-back and 3-back scores is significant for A' scores [*F*_(1.13)_= 36.073; *p* < 0.001], and for the Bias measure [*F*_(1.13)_ = 4.732; *p* = 0.049]. The mean RT shows a significant increase in the 3-back condition [*F*_(1.13)_ = 6.837; *p* = 0.021].

**Table 1:** **Means scores and standard deviations in the 1-back and 3-back tests**.

	**1-back (90 trials)**		**3-back (90 trials)**		***F*_(1.13)_**
	**Mean**	***SD***	**Mean**	***SD***	
A′	0.984	±0.017	0.849	±0.076	36.073^**^
B″d	−0.056	±0.835	0.550	±0.416	4.732^*^
RT	573.943	±105.100	661.164	±135.946	6.837^*^

** *p* < *0.01;*

* *p* < *0.05*.

*A′, sensitivity measure; B″d, decision criterion; RT, reaction time; SD, Standard Deviation*.

*In the 3-back, as compared to the 1-back, the subjects obtained significant lower scores in all the parameters considered*.

### Frequency analysis

**Figure 1:**
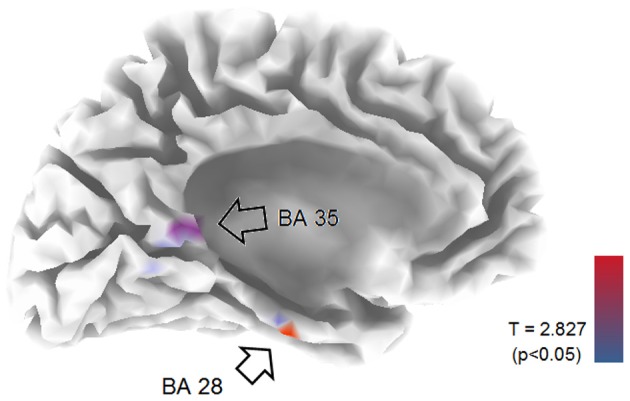
**LORETA representation of the mesial surface of the left hemisphere of the brain.** Arrows and colored spots indicate areas were statistically significant differences between 1-back and 3-back tasks were detected. BA, Brodmann areas. Graphic representation of the levels of significance, and threshold values (*T*), are reported in the lower right corner.

EEG recordings suitable for the analysis (that means, containing a sufficient amount of artifact-free EEG data) were obtained in all cases. The threshold for statistical significance, calculated by the sLORETA statistical tool, was *T* = 2.827 corresponding to *p* < 0.05. No significant differences were observed, between the 1-back and 3-back conditions, in the power of theta, alpha, beta, and gamma frequency bands. Statistically significant differences were observed in the delta frequency band: in the 3-back condition an increased power of delta activity was localized in a brain region corresponding to the Brodmann Area (BA) 28 in the left posterior entorhinal cortex (*T* = 3.112; *p* < 0.05) and in the BA 35 in the left perirhinal cortex in the parahippocampal gyrus (*T* = 2.876; *p* < 0.05) (Figure 1). No significant differences were observed in the right hemisphere. The mean relative power spectral values for each frequency band, in all scalp electrodes recorded, are reported in Table 2. The cortical areas where the most prominent modifications of spectral power were observed, even if not significant, are reported in Table 3.

**Table 2:** **Relative power spectral values (expressed as mean ± standard deviation) for each frequency band, in all scalp electrodes recorded**.

**1-back**	**Delta**		**Theta**		**Alpha**		**Beta**		**Gamma**		**3-back**	**Delta**		**Theta**		**Alpha**		**Beta**		**Gamma**	
	**Mean**	***SD***	**Mean**	***SD***	**Mean**	***SD***	**Mean**	***SD***	**Mean**	***SD***		**Mean**	***SD***	**Mean**	***SD***	**Mean**	***SD***	**Mean**	***SD***	**Mean**	***SD***
Fp1	7.8	5.1	7.5	3.0	9.0	3.5	16.9	4.7	58.7	9.0	Fp1	7.6	5.4	6.7	4.7	8.4	3.7	15.7	4.2	61.6	8.7
Fp2	7.9	5.7	7.9	3.9	9.8	3.7	16.3	4.2	58.0	11.7	Fp2	7.8	5.3	6.9	5.1	8.9	5.2	14.8	4.7	61.5	9.8
F7	7.7	5.3	7.8	3.5	8.4	3.1	15.7	4.9	60.4	10.4	F7	8.4	5.6	8.2	4.7	8.8	5.2	14.3	4.0	60.2	8.4
F3	7.7	5.1	8.1	3.4	8.4	4.0	15.9	4.8	59.9	8.5	F3	8.5	6.3	7.3	4.9	7.7	3.9	14.3	2.6	62.2	9.5
Fz	7.3	5.5	8.3	3.9	9.4	4.1	16.0	4.8	58.9	11.3	Fz	7.6	5.7	7.4	4.2	9.1	4.2	14.6	3.5	61.3	7.4
F4	7.3	4.7	8.1	3.7	8.8	3.2	16.2	4.7	59.6	6.8	F4	7.7	5.7	7.6	4.7	8.3	4.9	13.5	3.8	62.9	8.6
F8	6.8	4.3	7.8	3.2	8.6	4.1	15.7	4.2	61.1	7.9	F8	8.6	5.9	7.2	4.5	7.4	4.2	14.1	3.3	62.6	8.8
T3	7.5	5.5	8.0	4.0	9.0	3.3	16.1	5.2	59.5	11.8	T3	7.6	5.7	7.2	4.4	8.0	3.7	14.8	4.1	62.4	8.3
C3	7.3	5.0	7.8	3.8	8.5	3.4	15.8	5.9	60.6	8.4	C3	8.4	5.9	7.4	4.8	9.0	5.0	13.4	4.2	61.9	8.7
Cz	7.3	4.4	8.2	3.1	8.7	4.2	15.8	5.1	60.0	8.0	Cz	8.5	5.5	7.5	4.6	7.2	3.6	14.4	4.1	62.3	7.4
C4	7.0	5.0	8.0	3.9	9.3	3.1	16.3	5.2	59.4	10.4	C4	8.1	5.6	7.2	4.2	8.0	4.0	14.1	4.5	62.6	8.1
T4	7.1	5.2	7.9	3.8	8.7	3.8	15.8	6.5	60.5	9.5	T4	8.2	5.9	7.4	4.7	8.8	4.9	14.1	4.7	61.5	8.5
T5	7.5	4.9	7.6	3.7	8.9	4.0	16.5	5.7	59.5	9.3	T5	9.0	6.1	8.0	4.7	7.9	4.2	14.3	4.8	60.8	8.3
P3	6.9	4.9	8.0	4.0	9.0	3.7	16.8	5.3	59.3	10.6	P3	7.8	6.0	6.9	5.1	7.4	3.9	14.5	5.0	63.3	9.9
Pz	7.1	5.1	7.9	3.7	8.8	3.4	16.3	6.7	59.9	10.1	Pz	8.0	5.3	7.0	4.7	8.8	5.2	14.8	4.4	61.4	8.5
P4	7.3	4.9	7.7	3.9	8.5	3.3	16.6	5.2	60.0	9.0	P4	8.3	5.8	7.4	4.7	8.2	4.2	14.3	4.2	61.9	8.9
T6	6.9	5.1	7.6	3.4	9.0	3.8	16.2	3.4	60.2	10.0	T6	7.6	5.2	6.2	4.4	7.7	3.5	14.9	4.4	63.6	8.1
O1	7.7	5.9	7.9	3.7	9.7	3.3	16.7	5.7	58.0	11.3	O1	8.1	5.1	7.1	4.8	9.4	4.9	14.3	3.6	61.1	8.0
O2	7.3	5.0	7.9	3.1	8.5	3.8	16.9	6.1	59.4	9.3	O2	8.3	5.7	7.1	4.8	8.3	4.9	14.7	4.7	61.7	8.8

SD, Standard Deviation.

**Table 3:** **Cortical areas where the most prominent modifications of spectral power were observed (result of the non-parametric Fisher's permutation test are expressed ad *T* values; *T*-threshold for *p* < 0.05 is *T* = 2.827)**.

**Frequency band**	**Broadmann area**	**Cortical gyrus**	***T* score**
Delta	28	Parahippocampal gyrus	3.112^*^
	35	Parahippocampal gyrus	3.112^*^
Theta	30	Posterior cingulate	1.950
	29	Posterior cingulate	1.950
Alpha	29	Posterior cingulate	1.470
	30	Posterior cingulate	1.470
Beta	20	Inferior temporal	1.190
	36	Uncus	1.190
Gamma	41	Superior temporal	1.210
	13	Superior temporal	1.210

* *p* < *0.05*.

Comparison of power spectra between group of scalp electrodes, for each frequency band, showed no significant differences in the frequency bands theta, alpha, beta, and gamma. In the delta ban, an increase of delta power in the parieto-temporal leads was observed (mean delta power 1-back: 21.2 ± 22.3; 3-back: 44.7 ± 76.3 μV^2^/Hz; *U*-test = 3499.5: *p* = 0.001). The comparison between right parieto-temporal vs. left parieto-temporal leads showed a significant difference in the 3-back condition (Left: 6.9 ± 6.7 μV^2^/Hz; Right: 4.2 ± 6.4 μV^2^/Hz; *U*-test = 1388; *p* < 0.001), but not in 1-back (Left: 7.0 ± 6.5 μV^2^/Hz; Right: 8.5 ± 6.7 μV^2^/Hz; *U*-test = 1090; *p* = 0.063).

## Discussion

The aim of this study was to measure modifications in EEG cortical activity during a WM task, namely *n*-back, of increasing difficulty. In this respect, the main finding was that, as compared to the easy 1-back task, the more complex 3-back task induced significant modifications in the EEG activity of left mesial temporal structures in the delta frequency band. The left hemisphere and the para-hippocampal cortices were selectively involved, in particular the BA 28 and 35. This finding, obtained by applying the sLORETA source modeling, was also substantially confirmed by comparing scalp EEG power spectra in the delta frequency band.

The results of the present study are not consistent with those reported in previous research; in particular, we did not observe significant power modifications involving the alpha and theta bands. Previous papers (Gevins et al., 1997; Gevins and Smith, 2000; Deiber et al., 2007; Lei and Roetting, 2011; Palomaki et al., 2012) described EEG changes linked to the complexity of the *n*-back task that occurred prevalently in the alpha and theta frequency bands, but the possible change in delta frequency has not been considered. Furthermore, EEG modification described in previous studies, occurred especially in frontal and parietal lobs. The involvement of these areas has been documented by other studies using different methods (Owen et al., 2005; Brookes et al., 2011).

Firstly our results indicate that the only significant modification induced by the increased complexity of the task occur in the mesial temporal lobe structure. This is consistent with recent data (Axmacher et al., 2007, 2010) which indicate that mesial temporal cortices play an import role for the WM tasks. The role of mesial temporal lobe in the WM system, however, is still matter of debate (Jeneson et al., 2010, 2012; Jeneson and Squire, 2011; Bergmann et al., 2012; Stretton et al., 2012).

In a recent study, Axmacher et al. (2010) investigated the relationship between different oscillatory patterns of hippocampal neurons, and their association with performance in WM tasks. These authors found that the maintenance of items in WM is associated with changes in hippocampal functioning, and that these changes involve prominently the amplitude of beta/gamma and theta oscillations. With respect to these findings, our study confirms that increasing complexity in WM tasks induces modifications of EEG activity in the mesial temporal regions; nevertheless, we measured modifications in the low-frequency delta band. This could in part be related to the different approach to EEG analysis: we adopted frequency analysis, whereas the cited Authors applied a cross-frequency coupling analysis that is, a connectivity analysis. Moreover, the authors (Axmacher et al., 2010) performed intracranial EEG recordings: in particular, the analysis of fast-frequency EEG rhythms (such as gamma activity) can be hardly performed in scalp EEG study.

We hypothesize that our results could be a consequence of the involvement of specific brain areas in memory functions. It is known that hippocampal and parahippocampal cortices predominantly show low-frequency (theta and delta) activities which are coupled, during memory tasks, with faster EEG rhythms (gamma) generated in the neocortex (Wang, 2010). In an intracranial recording study, Mormann and coworkers (2008) observed independent delta and theta rhythms in sub-regions of the human MTL. According to the Authors, “the interaction of these rhythms could represent the temporal basis for the information processing required for mnemonic encoding and retrieval.” The sLORETA analysis, within the limitation of a surface EEG recording, allowed to detect, during the 3-back task, the selective increase of delta power in a portion of the perhirinal and entorhinal cortices, corresponding to the BA 35 and 28. Perirhinal cortex contributes to mediate the dialog between hippocampus and neocortex (Wang, 2010). Entorhinal cortex contains neurons which are selectively activated during tasks which require simultaneously spatial navigation and memory (Quirk et al., 1992; Frank et al., 2000; Hafting et al., 2005; Hori et al., 2005; Eichenbaum et al., 2007; Solstad et al., 2008; Doeller et al., 2010; Jacobs et al., 2010). This finding, emerging from studies performed in animal models (Quirk et al., 1992; Frank et al., 2000; Hafting et al., 2005; Hori et al., 2005; Solstad et al., 2008), has received several confirmations in human studies (Eichenbaum et al., 2007; Doeller et al., 2010; Jacobs et al., 2010). Therefore, it could be speculated that the activation of this cortex during the 3-back test could be related to the high complexity of the task. This increasing complexity could require the application of a more complex mental strategy, including the construction of a space model of the incoming stimuli. The ability to dispose the stimuli in a spatial framework could help the WM system to analyze them and to detect the target. The defined left-side localization of the activation seen in our study might be due to the use of verbal stimuli for the *n*-back task (Smith and Jonides, 1997; Nystrom et al., 2000; Owen et al., 2005); alternatively, the lateralization could be directly due to the increased level of difficulty of the task (Ross and Segalowitz, 2000). Moreover, the hit trials were associated with a motor response: the activation of motor areas could introduce a lateralization bias in the EEG spectra, which could explain the left hemisphere activation shown by sLORETA. As concerns, it must be specified that all subjects enrolled in the study were right-handed and pressed the response bar with their right hand.

Further explanations for the discrepancy between our results and those reported in literature are possible. In a study by Corsi-Cabrera et al, power spectra from wake and sleep I in healthy adults subjects were submitted to Principal Component Analyses to investigate which frequencies covaried together (Corsi-Cabrera et al., 2000). The results indicated that slow wave activity can oscillate at higher frequencies, up to 8 Hz; interestingly, no theta band was independently identified: according to the Authors, this suggested either that “delta and theta oscillations are two rhythms under the same global influence, or that the traditional division of theta band in the human cortical EEG is artificial” (Corsi-Cabrera et al., 2000).

Stimulus expectation could also play a role: in the 3-back protocol, a Contingent Negative Variation (CNV) potential could in theory develop between the first stimulus and the target stimulus, and it could bias the results. Nevertheless, the CNV is localized over the frontal and the motor cortical areas (Rohrbaugh et al., 1976), and is not on recorded on the mesial temporal regions where we observed the significant differences between 1-back and 3-back tasks.

The present study has some limitations. The first is the use of scalp EEG recordings, which have an intrinsic limit in space resolution, particularly in the identification of deep subcortical sources. A further limit is in the montage applied, which is the one used in standard EEG recording. It is known that spatial resolution of EEG sources increases with the number of electrodes, and therefore high-density recordings are reliable in the esteem of EEG rhythms source analysis. Moreover, magnetoencephalographic (MEG) recordings are even more reliable in identifying deep EEG sources. Cohen et al. (1990) performed a study in which they evaluated the localization of signal sources by means of scalp EEG and MEG. In this study, they used as signal sources intra-cerebral electrodes implanted for seizure monitoring; the signal was a weak current pulse which was passed in the implanted electrodes. In this study, they demonstrated that a scalp EEG array of 16 electrodes allowed source localization with an average error = 10 mm; this accuracy was not different from that obtained with MEG recordings (average error = 8 mm). The same kind of limitation, obviously, is reflected by the sLORETA software, which is, by definition a Low Resolution electric source analysis software.

In conclusion, our findings suggest that the 3-back tasks induce an increase of delta oscillations in the mesial temporal lobe structures that allow to sustain the increased complexity of the task.

### Conflict of interest statement

The authors declare that the research was conducted in the absence of any commercial or financial relationships that could be construed as a potential conflict of interest.

## References

[B1] Andres P. (2003). Frontal cortex as the central executive of working memory: time to revise our view. Cortex 39, 871–895 14584557 10.1016/s0010-9452(08)70868-2

[B2] Axmacher N. Henseler M. M. Jensen O. Weinreich I. Elger C. E. Fell J. (2010). Cross-frequency coupling supports multi-item working memory in the human hippocampus. Proc. Natl. Acad. Sci. U.S.A. 107, 3228–3233 10.1073/pnas.0911531107 20133762 PMC2840289

[B3] Axmacher N. Mormann F. Fernandez G. Cohen M. X. Elger C. E. Fell J. (2007). Sustained neural activity patterns during working memory in the human medial temporal lobe. J. Neurosci. 27, 7807–7816 10.1523/JNEUROSCI.0962-07.2007 17634374 PMC6672876

[B4] Baddeley A. (2000). The episodic buffer: a new component of working memory? Trends Cogn. Sci. 4, 417–423 10.1016/S1364-6613(00)01538-2 11058819

[B5] Baddeley A. (2010). Working memory. Curr. Biol. 20, R136–R140 20178752 10.1016/j.cub.2009.12.014

[B6] Baddeley A. (2011). Working memory: theories, models, and controversies. Annu. Rev. Psychol. 63, 1–29 10.1146/annurev-psych-120710-100422 21961947

[B7] Bergmann H. C. Rijpkema M. Fernandez G. Kessels R. P. (2012). Distinct neural correlates of associative working memory and long-term memory encoding in the medial temporal lobe. Neuroimage 63, 989–997 10.1016/j.neuroimage.2012.03.047 22484305

[B8] Bledowski C. Kaiser J. Rahm B. (2010). Basic operations in working memory: contributions from functional imaging studies. Behav. Brain Res. 214, 172–179 10.1016/j.bbr.2010.05.041 20678984

[B9] Braver T. S. Cohen J. D. Nystrom L. E. Jonides J. Smith E. E. Noll D. C. (1997). A parametric study of prefrontal cortex involvement in human working memory. Neuroimage 5, 49–62 10.1006/nimg.1996.0247 9038284

[B10] Brookes M. J. Wood J. R. Stevenson C. M. Zumer J. M. White T. P. Liddle P. F. (2011). Changes in brain network activity during working memory tasks: a magnetoencephalography study. Neuroimage 55, 1804–1815 10.1016/j.neuroimage.2010.10.074 21044687 PMC6485426

[B11] Chatham C. H. Herd S. A. Brant A. M. Hazy T. E. Miyake A. O'Reilly R. (2011). From an executive network to executive control: a computational model of the n-back task. J. Cogn. Neurosci. 23, 3598–3619 10.1162/jocn_a_00047 21563882 PMC3269304

[B12] Cohen D. Cuffin B. N. Yunokuchi K. Maniewski R. Purcell C. Cosgrove G. R. (1990). MEG versus EEG localization test using implanted sources in the human brain. Ann. Neurol. 28, 811–817 10.1002/ana.410280613 2285267

[B13] Collette F. Salmon E. Van Der Linden M. Chicherio C. Belleville S. Degueldre C. (1999). Regional brain activity during tasks devoted to the central executive of working memory. Brain Res. Cogn. Brain Res. 7, 411–417 10.1016/S0926-6410(98)00045-7 9838207

[B14] Conway A. R. Kane M. J. Bunting M. F. Hambrick D. Z. Wilhelm O. Engle R. W. (2005). Working memory span tasks: a methodological review and user's guide. Psychon. Bull. Rev. 12, 769–786 16523997 10.3758/bf03196772

[B15] Corsi-Cabrera M. Guevara M. A. Del Rio-Portilla Y. Arce C. Villanueva-Hernandez Y. (2000). EEG bands during wakefulness, slow-wave and paradoxical sleep as a result of principal component analysis in man. Sleep 23, 738–744 11007440

[B16] Deiber M. P. Missonnier P. Bertrand O. Gold G. Fazio-Costa L. Ibanez V. (2007). Distinction between perceptual and attentional processing in working memory tasks: a study of phase-locked and induced oscillatory brain dynamics. J. Cogn. Neurosci. 19, 158–172 10.1162/jocn.2007.19.1.158 17214572

[B17] D'Esposito M. Detre J. A. Alsop D. C. Shin R. K. Atlas S. Grossman M. (1995). The neural basis of the central executive system of working memory. Nature 378, 279–281 10.1038/378279a0 7477346

[B18] Doeller C. F. Barry C. Burgess N. (2010). Evidence for grid cells in a human memory network. Nature 463, 657–661 10.1038/nature08704 20090680 PMC3173857

[B19] Druzgal T. J. D'Esposito M. (2001). Activity in fusiform face area modulated as a function of working memory load. Brain Res. Cogn. Brain Res. 10, 355–364 10.1016/S0926-6410(00)00056-2 11167061

[B20] Eichenbaum H. Yonelinas A. P. Ranganath C. (2007). The medial temporal lobe and recognition memory. Annu. Rev. Neurosci. 30, 123–152 10.1146/annurev.neuro.30.051606.094328 17417939 PMC2064941

[B21] Frank L. M. Brown E. N. Wilson M. (2000). Trajectory encoding in the hippocampus and entorhinal cortex. Neuron 27, 169–178 10.1016/S0896-6273(00)00018-0 10939340

[B22] Garavan H. Ross T. J. Li S. J. Stein E. A. (2000). A parametric manipulation of central executive functioning. Cereb. Cortex 10, 585–592 10.1093/cercor/10.6.585 10859136

[B23] Gevins A. Smith M. E. (2000). Neurophysiological measures of working memory and individual differences in cognitive ability and cognitive style. Cereb. Cortex 10, 829–839 10.1093/cercor/10.9.829 10982744

[B24] Gevins A. Smith M. E. McEvoy L. Yu D. (1997). High-resolution EEG mapping of cortical activation related to working memory: effects of task difficulty, type of processing, and practice. Cereb. Cortex 7, 374–385 10.1093/cercor/7.4.374 9177767

[B25] Grave De Peralta-Menendez R. Gonzalez-Andino S. L. (1998). A critical analysis of linear inverse solutions to the neuroelectromagnetic inverse problem. IEEE Trans. Biomed. Eng. 45, 440–448 10.1109/10.664200 9556961

[B26] Grave De Peralta Menendez R. Gonzalez Andino S. L. Morand S. Michel C. M. Landis T. (2000). Imaging the electrical activity of the brain: ELECTRA. Hum. Brain Mapp. 9, 1–12 10.1002/(SICI)1097-0193(2000)9:1<1::AID-HBM1>3.0.CO;2-# 10643725 PMC6871828

[B27] Hafting T. Fyhn M. Molden S. Moser M. B. Moser E. I. (2005). Microstructure of a spatial map in the entorhinal cortex. Nature 436, 801–806 10.1038/nature03721 15965463

[B28] Hamalainen M. (1995). Discrete and distributed source estimates, in ISBET Newsletter, No 6, ed Skrandies W. (Giessen, Germany), 9–12

[B29] Honey G. D. Bullmore E. T. Sharma T. (2002). De-coupling of cognitive performance and cerebral functional response during working memory in schizophrenia. Schizophr. Res. 53, 45–56 11728837 10.1016/s0920-9964(01)00154-2

[B30] Hori E. Nishio Y. Kazui K. Umeno K. Tabuchi E. Sasaki K. (2005). Place-related neural responses in the monkey hippocampal formation in a virtual space. Hippocampus 15, 991–996 10.1002/hipo.20108 16108028

[B31] Jacobs J. Kahana M. J. Ekstrom A. D. Mollison M. V. Fried I. (2010). A sense of direction in human entorhinal cortex. Proc. Natl. Acad. Sci. U.S.A. 107, 6487–6492 10.1073/pnas.0911213107 20308554 PMC2851993

[B32] Jansma J. M. Ramsey N. F. Coppola R. Kahn R. S. (2000). Specific versus nonspecific brain activity in a parametric N-back task. Neuroimage 12, 688–697 10.1006/nimg.2000.0645 11112400

[B33] Jeneson A. Mauldin K. N. Squire L. R. (2010). Intact working memory for relational information after medial temporal lobe damage. J. Neurosci. 30, 13624–13629 10.1523/JNEUROSCI.2895-10.2010 20943903 PMC2975488

[B34] Jeneson A. Squire L. R. (2011). Working memory, long-term memory, and medial temporal lobe function. Learn. Mem. 19, 15–25 10.1101/lm.024018.111 22180053 PMC3246590

[B35] Jeneson A. Wixted J. T. Hopkins R. O. Squire L. R. (2012). Visual working memory capacity and the medial temporal lobe. J. Neurosci. 32, 3584–3589 10.1523/JNEUROSCI.6444-11.2012 22399780 PMC3349278

[B36] Kane M. J. Engle R. W. (2002). The role of prefrontal cortex in working-memory capacity, executive attention, and general fluid intelligence: an individual-differences perspective. Psychon. Bull. Rev. 9, 637–671 12613671 10.3758/bf03196323

[B37] Kreiter A. K. Singer W. (1992). Oscillatory neuronal responses in the visual cortex of the awake macaque monkey. Eur. J. Neurosci. 4, 369–375 12106363 10.1111/j.1460-9568.1992.tb00884.x

[B38] Lei S. Roetting M. (2011). Influence of task combination on EEG spectrum modulation for driver workload estimation. Hum. Factors 53, 168–179 21702334 10.1177/0018720811400601

[B39] Linden D. E. (2007). The working memory networks of the human brain. Neuroscientist 13, 257–267 10.1177/1073858406298480 17519368

[B40] McEvoy L. K. Smith M. E. Gevins A. (1998). Dynamic cortical networks of verbal and spatial working memory: effects of memory load and task practice. Cereb. Cortex 8, 563–574 10.1093/cercor/8.7.563 9823478

[B41] Mitchell D. J. McNaughton N. Flanagan D. Kirk I. J. (2008). Frontal-midline theta from the perspective of hippocampal “theta.” Prog. Neurobiol. 86, 156–185 10.1016/j.pneurobio.2008.09.005 18824212

[B42] Mormann F. Osterhage H. Andrzejak R. G. Weber B. Fernandez G. Fell J. (2008). Independent delta/theta rhythms in the human hippocampus and entorhinal cortex. Front. Hum. Neurosci. 2:3 10.3389/neuro.09.003.2008 18958204 PMC2525973

[B43] Muller N. G. Knight R. T. (2006). The functional neuroanatomy of working memory: contributions of human brain lesion studies. Neuroscience 139, 51–58 10.1016/j.neuroscience.2005.09.018 16352402

[B44] Murphy T. H. Blatter L. A. Wier W. G. Baraban J. M. (1992). Spontaneous synchronous synaptic calcium transients in cultured cortical neurons. J. Neurosci. 12, 4834–4845 1361198 10.1523/JNEUROSCI.12-12-04834.1992 PMC6575780

[B45] Nichols T. E. Holmes A. P. (2002). Nonparametric permutation tests for functional neuroimaging: a primer with examples. Hum. Brain Mapp. 15, 1–25 10.1002/hbm.1058 11747097 PMC6871862

[B46] Nystrom L. E. Braver T. S. Sabb F. W. Delgado M. R. Noll D. C. Cohen J. D. (2000). Working memory for letters, shapes, and locations: fMRI evidence against stimulus-based regional organization in human prefrontal cortex. Neuroimage 11, 424–446 10.1006/nimg.2000.0572 10806029

[B47] Onton J. Delorme A. Makeig S. (2005). Frontal midline EEG dynamics during working memory. Neuroimage 27, 341–356 10.1016/j.neuroimage.2005.04.014 15927487

[B48] Owen A. M. McMillan K. M. Laird A. R. Bullmore E. (2005). N-back working memory paradigm: a meta-analysis of normative functional neuroimaging studies. Hum. Brain Mapp. 25, 46–59 10.1002/hbm.20131 15846822 PMC6871745

[B49] Palomaki J. Kivikangas M. Alafuzoff A. Hakala T. Krause C. M. (2012). Brain oscillatory 4-35 Hz EEG responses during an n-back task with complex visual stimuli. Neurosci. Lett. 516, 141–145 10.1016/j.neulet.2012.03.076 22490880

[B50] Pascual-Marqui R. D. Michel C. M. Lehmann D. (1994). Low resolution electromagnetic tomography: a new method for localizing electrical activity in the brain. Int. J. Psychophysiol. 18, 49–65 10.1016/0167-8760(84)90014-X 7876038

[B51] Payne L. Kounios J. (2009). Coherent oscillatory networks supporting short-term memory retention. Brain Res. 1247, 126–132 10.1016/j.brainres.2008.09.095 18976639 PMC2706422

[B52] Pesonen M. Hamalainen H. Krause C. M. (2007). Brain oscillatory 4-30 Hz responses during a visual n-back memory task with varying memory load. Brain Res. 1138, 171–177 10.1016/j.brainres.2006.12.076 17270151

[B53] Quirk G. J. Muller R. U. Kubie J. L. Ranck J. B. Jr. (1992). The positional firing properties of medial entorhinal neurons: description and comparison with hippocampal place cells. J. Neurosci. 12, 1945–1963 1578279 10.1523/JNEUROSCI.12-05-01945.1992 PMC6575876

[B54] Ragland J. D. Turetsky B. I. Gur R. C. Gunning-Dixon F. Turner T. Schroeder L. (2002). Working memory for complex figures: an fMRI comparison of letter and fractal n-back tasks. Neuropsychology 16, 370–379 12146684 PMC4332798

[B55] Ravizza S. M. Behrmann M. Fiez J. A. (2005). Right parietal contributions to verbal working memory: spatial or executive? Neuropsychologia 43, 2057–2067 10.1016/j.neuropsychologia.2005.03.014 15885716

[B56] Rohrbaugh J. W. Syndulko K. Lindsley D. B. (1976). Brain wave components of the contingent negative variation in humans. Science 191, 1055–1057 10.1126/science.1251217 1251217

[B57] Ross P. Segalowitz S. J. (2000). An EEG coherence test of the frontal dorsal versus ventral hypothesis in N-back working memory. Brain Cogn. 43, 375–379 10857729

[B58] Salmon E. Van Der Linden M. Collette F. Delfiore G. Maquet P. Degueldre C. (1996). Regional brain activity during working memory tasks. Brain 119(Pt 5), 1617–1625 10.1093/brain/119.5.1617 8931584

[B59] Schlosser R. G. Wagner G. Sauer H. (2006). Assessing the working memory network: studies with functional magnetic resonance imaging and structural equation modeling. Neuroscience 139, 91–103 10.1016/j.neuroscience.2005.06.037 16324797

[B60] Smith E. E. Jonides J. (1997). Working memory: a view from neuroimaging. Cogn. Psychol. 33, 5–42 10.1006/cogp.1997.0658 9212720

[B61] Solstad T. Boccara C. N. Kropff E. Moser M. B. Moser E. I. (2008). Representation of geometric borders in the entorhinal cortex. Science 322, 1865–1868 10.1126/science.1166466 19095945

[B62] Stretton J. Winston G. Sidhu M. Centeno M. Vollmar C. Bonelli S. (2012). Neural correlates of working memory in Temporal Lobe Epilepsy—An fMRI study. Neuroimage 60, 1696–1703 10.1016/j.neuroimage.2012.01.126 22330313 PMC3677092

[B63] Wang X. J. (2010). Neurophysiological and computational principles of cortical rhythms in cognition. Physiol. Rev. 90, 1195–1268 10.1152/physrev.00035.2008 20664082 PMC2923921

